# Supplementary material for: The interactome of intact mitochondria by cross-linking mass spectrometry provides evidence for coexisting respiratory supercomplexes
Source: Mol Cell Proteomics. 2017 Dec 8;17(2):216–32. doi: 10.1074/mcp.RA117.000470 (PMC5795388; doi:10.1074/mcp.RA117.000470)
Supplement: Supplemental Data [file supp_RA117.000470_133922_0_supp_23978_fzffwf.zip › spectra_annotation/mito_DR_spectra_annotation/66-1-9-1-2-1.pdf]

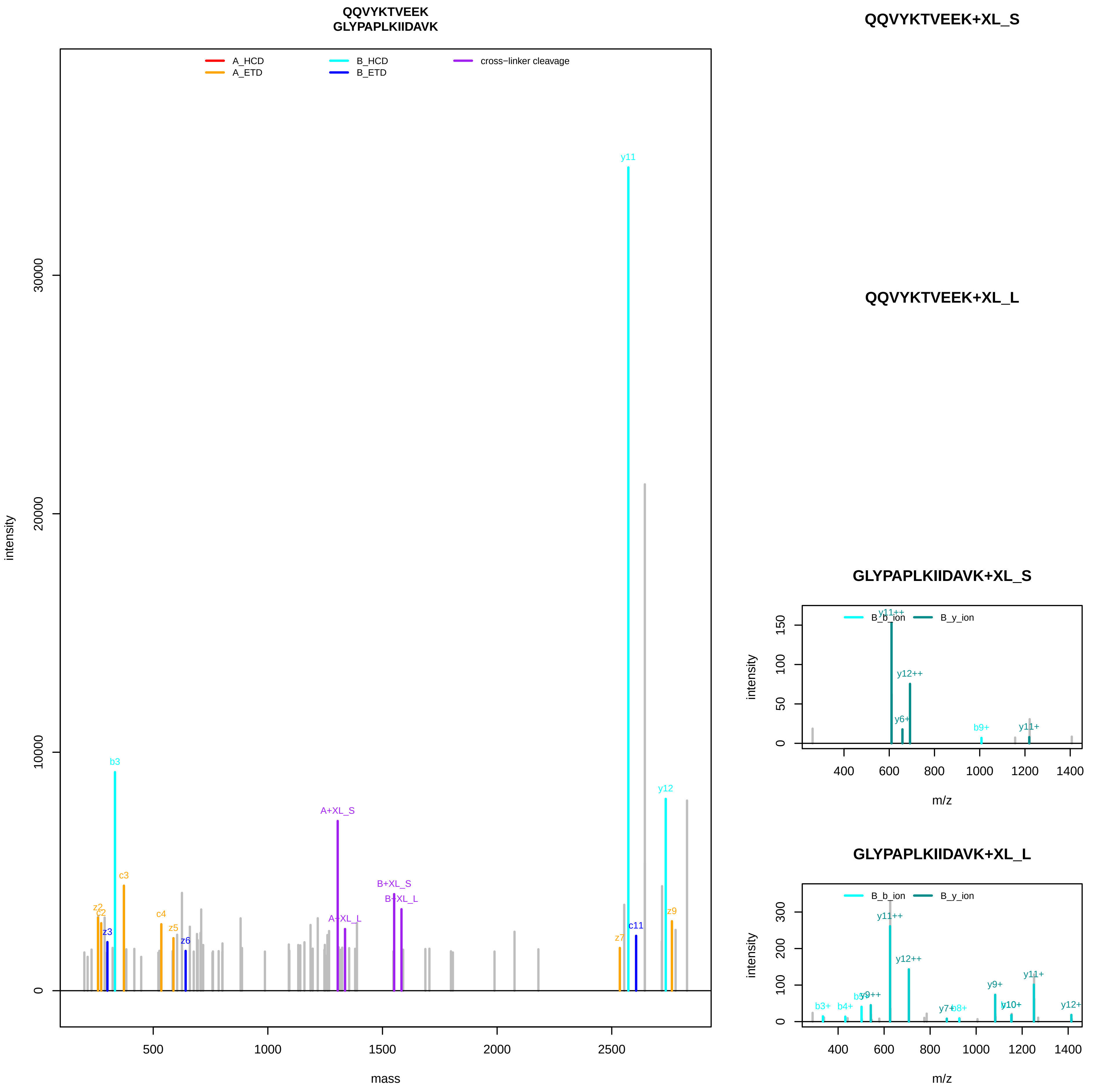
QQVYKTVEEK
GLYPAPLKIIDAVK
A_HCD
A_ETD
B_HCD
B_ETD
cross−linker cleavage
intensity
mass
y11
b3
y12
c3
z2
c2
z3
c4
z5
z6
A+XL_S
B+XL_S
B+XL_L
A+XL_L
z7
c11
z9
QQVYKTVEEK+XL_S
QQVYKTVEEK+XL_L
GLYPAPLKIIDAVK+XL_S
B_b_ion
B_y_ion
y11++
y12++
y6+
b9+
y11+
m/z
GLYPAPLKIIDAVK+XL_L
b3+
b4+
y9++
y7+
b8+
y9+
y10+
y12+
